# Bovine milk-derived extracellular vesicles reduce oxidative stress and ferroptosis induced by *Klebsiella pneumoniae* in bovine mammary epithelial cells

**DOI:** 10.1186/s40104-025-01151-7

**Published:** 2025-02-14

**Authors:** Bingchun Liang, Yindi Xiong, Eduardo R. Cobo, John Kastelic, Xiaofang Tong, Bo Han, Jian Gao

**Affiliations:** 1 Department of Clinical Veterinary Medicine, College of Veterinary Medicine, China Agricultural University, Yuanmingyuan West Road, Beijing, 100193 China; 2 Faculty of Veterinary Medicine, University of Calgary, Calgary, AB T2N 4N1 Canada

**Keywords:** Bovine mammary epithelial cells, Extracellular vesicles, Ferroptosis, *Klebsiella pneumoniae*, Oxidative stress

## Abstract

**Background:**

Ferroptosis is characterized by increased production of reactive oxygen species (ROS) and membrane lipid peroxidation that can exacerbate inflammatory damage. Extracellular vesicles (EVs) isolated from bovine milk have many biological functions, including antioxidant properties. However, the role of EVs on *Klebsiella pneumoniae*-induced ferroptosis and oxidative stress in bovine mammary epithelial cells (bMECs) and murine mammary tissue is unclear. In this study, EVs were isolated from bovine colostrum, mature milk and clinical mastitis milk (defined as C-EVs, M-EVs and CM-EVs, respectively) and assessed by transmission electron microscopy, Western blot and transcriptome sequencing. Effects of EVs on *K. pneumoniae*-induced ferroptosis and oxidative stress in bMECs were evaluated with immunofluorescence and Western blot.

**Results:**

In bMECs, infection with *K. pneumoniae* induced oxidative stress, decreasing protein expression of Nrf2, Keap1 and HO-1 plus SOD activity, and increasing ROS concentrations. However, protein expression of GPX4, ACSL4 and S100A4 in bMECs, all factors that regulate ferroptosis, was downregulated by *K. pneumoniae*. Furthermore, this bacterium compromised tight junctions in murine mammary tissue, with low expression of ZO-1 and Occludin, whereas protein expression of Nrf2 and GPX4 was also decreased in mammary tissue. Adding C-EVs, M-EVs or CM-EVs reduced oxidative stress and ferroptosis in *K. pneumoniae*-infected bMECs in vitro and murine mammary tissues in vivo.

**Conclusion:**

In conclusion, all 3 sources of milk-derived EVs alleviated oxidative stress and ferroptosis in *K. pneumoniae*-infected bMECs and mammary tissues.

**Supplementary Information:**

The online version contains supplementary material available at 10.1186/s40104-025-01151-7.

## Introduction

Extracellular vesicles (EVs) are membrane-bound structures secreted by cells; they contain various bioactive components, including proteins, lipids, nucleic acids, and both coding and non-coding RNA derived from donor cells. These bioactive components are released by exocytosis, exert paracrine actions on surrounding tissues, and participate in many physiological and pathological processes [1]. Bovine milk, including colostrum and mature milk, are rich in EVs, with colostrum containing significantly more EVs than mature milk [2]. The EVs from bovine milk have various biological functions, including enhancing antioxidant capacity and immune response, plus promoting angiogenesis, wound healing, and repair of intestinal mucosal immune barriers [3–6]. Milk from cows with mastitis also contains EVs, but whether their biological functions are similar to EVs from normal milk is unknown [7, 8].

Bovine mastitis caused by pathogenic microorganisms is usually accompanied by increased oxidative stress. In a previous epidemiological investigation, we reported that *Klebsiella pneumoniae* is one of the most common pathogens causing clinical mastitis on large dairy farms in China, with an isolation rate of 13.0%, second only to *Escherichia coli* (14.4%) [9]. However, compared to *E. coli*, clinical signs of *K. pneumoniae* caused mastitis are generally more severe, milk loss occurs earlier, the duration is longer, and damage to mammary tissue is more serious [9, 10]. In bovine mammary epithelial cells (bMECs), infection with *K. pneumoniae* increased reactive oxygen species (ROS) and damaged mitochondria [11]. Nuclear factor erythroid 2-related factor 2 (Nrf2) has a key role in the antioxidant activity of cells; it is an important regulator of iron promotion and contributes to other vital pathways, including lipid metabolism, iron homeostasis and energy metabolism, which modulate ferroptosis [12, 13]. Under normal oxygen conditions, Nrf2 binds to Kelch-like ECH-associated protein 1 (Keap1) and is inactivated by ubiquitination and degradation in the proteasome [14]. However, under conditions of oxidative stress, cysteine residues (e.g., Cys151, Cys273, Cys288) that are oxidized or covalently modified can change the conformation of Keap1; consequently, inhibition and ubiquitination of Nrf2 can be reduced and Nrf2 released from Keap1 and rapidly transferred to the nucleus, where it interacts with antioxidant response elements in the promoter region to maintain cellular REDOX homeostasis [15, 16]. Similarly, heme oxygenase-1 (HO-1) can be further regulated by Nrf2 in cascade reactions of oxidant stress [17, 18].

Ferroptosis is a form of cell death initiated by iron-dependent phospholipid peroxidation and regulated by pathways involved in maintaining iron balance and managing oxidative stress. Glutathione peroxidase 4 (GPX4) has a crucial role in regulating ferroptosis by halting the chain reaction of lipid peroxidation, thereby preventing ferroptosis [19, 20]. *Mycobacterium tuberculosis* inhibits transcription and expression of GPX4 in host cells, ultimately inducing iron death in host cells and promoting pathogenicity [21]. S100 calcium binding protein A4 (S100A4) is a potent factor in resisting ferroptosis, as it produces metabolites with free radical-trapping antioxidant activity [22, 23]. Moreover, ferroptotic signaling is related to serine protease thrombin promoting arachidonic acid mobilization and esterification of acyl-CoA synthetase long-chain family member 4 (ACSL4) [24].

Infection of bMECs with *K. pneumoniae* can induce oxidative stress and apoptosis [11], but it is unclear whether ferroptosis occurs in *K. pneumoniae*-infected bMECs. Furthermore, the role of milk-derived EVs in *K. pneumoniae* infection of bMECs is also unclear. Therefore, EVs were isolated from colostrum, mature milk and clinical mastitis milk (defined as C-EVs, M-EVs and CM-EVs, respectively), and their microRNA (miRNA) cargo were analyzed using small RNA-seq. The objective was to determine effects of EVs on *K. pneumoniae*-induced oxidative stress and ferroptosis in bMECs in vitro and murine mammary tissue in vivo.

## Materials and methods

### Statement of ethics

This study was conducted in full compliance with the Beijing Municipality Guidelines on the Review of Welfare and Ethics of Laboratory Animals. Animal use was approved by the Beijing Municipality Administration Office of Laboratory Animals and the China Agricultural University Animal Ethics Committee (protocol AW62804202-2-6).

### Milk collection

Milk samples were collected from Holstein cows on a dairy farm in Hebei, using standard procedures to minimize contamination, and stored at −80 °C. Colostrum was collected immediately after calving, mature milk was collected mid-lactation and clinical mastitis milk was collected from mastitis cases. Each group contained 3 cows that were similar in age, weight, and parity.

### Isolation and characterization of extracellular vesicles (EVs)

For all milk samples, 50 mL was centrifuged at 5,000 × *g* for 30 min at 4 °C, the supernatant collected and centrifuged at 12,000 × *g* for 30 min at 4 °C. Then, the supernatant was collected and centrifuged at 100,000 × *g* for 1 h at 4 °C (Optima XPN-100 ultracentrifuge with Type SW32 Ti swing rotor, Beckman, Waltham, MA, USA). Next, the supernatant was centrifuged at 125,000 × *g* for 1 h at 4 °C, and the pellet was re-suspended in phosphate-buffered saline (PBS) and washed by ultracentrifugation at 4 °C at 125,000 × *g* for 1 h. The EVs, including C-EVs, M-EVs and CM-EVs were transferred onto 0.22-μm filters (Merck KGaA, Darmstadt, Germany) and centrifuged twice at 3,000 × *g* for 30 min. Protein concentrations of EVs were measured with a BCA protein assay kit (Beyotime, Shanghai, China). Size distribution and particle concentrations of EVs were assessed with a PMX120 ZetaView (Particle Metrix, Wildmoos, Germany). Samples were diluted in PBS at 4 °C. For morphology, 10 μL of EVs was incubated for 1 min on a formvar film-coated FF200-Cu grid (200 mesh, CU), negative-stained with uranyl acetate for 1 min and imaged with an FHT7800 transmission electron microscope (TEM; Hitachi, Tokyo, Japan).

### MiRNA sequencing of EVs

Total RNA was extracted from EVs using TRIzol (Invitrogen, Thermo Fisher Scientific, Inc., Carlsbad, CA, USA), purified by 2 phenol–chloroform treatments, and then treated with RQ1 DNase (Promega, Madison, WI, USA) to remove DNA. Smartspec Plus (BioRad, Hercules, CA, USA) was used to assess RNA quality and quantity. Total RNAs from each sample were used for miRNA cDNA library preparation with a Balancer NGS Library Preparation kit (Gnomegen, San Diego, CA, USA), according to the manufacturer’s protocol, prior to directional RNA-seq library preparation. These miRNA libraries were applied to HiSeq 2500 (Illumina, Inc., San Diego, CA, USA) for 150 bp pair-end sequencing.

### Bacterial challenge

*K. pneumoniae* strain (KPHB132952), recovered from a bovine clinical mastitis milk sample, was used to induce infection of bMECs and murine mammary gland tissue. The strain belonged to the capsule serotype (K57) and was stored at −80 °C. The *K. pneumoniae* suspensions were prepared from frozen stocks on a Luria–Bertani nutrient culture medium and incubated in a shaker (37 °C, 220 r/min) for 16 h. Thereafter, the strain was cultivated to the logarithmic growth phase before being used to infect bMECs in vitro and murine mammary gland tissue in vivo.

### Culture and infection of bMECs

MAC-T cells (Jingma Biological Technology Co., Ltd., Shanghai, China) were cultured in DMEM medium supplemented with 10% fetal bovine serum, penicillin (100 U/mL) and streptomycin (100 U/mL), then incubated in cell culture plates in 5% CO_2_ at 37 °C. After cell passages 2–8, 6-well plates were seeded (1 × 10^5^ cells/well) and cultured for 24 h, then infected with *K. pneumoniae* at a 5:1 multiplicity of infection (ratio of *K. pneumoniae* to cells). Half of the plates were treated with EVs (250 µg/mL) for 6 or 9 h at the cell-growth stage of 80% confluence.

### Transmission electron microscopy

Cells were infected as described above. At 6 or 9 h after infection, cells were washed 3 times with PBS, then fixed with 2.5% glutaraldehyde for 4 h and post-fixed in 0.5% osmium tetroxide for 2 h. Ethanol gradient dehydration was the first step, followed by acetone. Thereafter, samples were embedded in resin and thin slices (100 nm) were cut with a glass knife. Copper grids covered with the sections were stained with 2% uranyl acetate and lead citrate and observed with TEM (H7650, Tokyo, Japan) at an accelerating voltage of 80 kV.

### Intracellular SOD activity and ROS content

Cells were treated as described above. At 6 or 9 h after infection, cell culture medium was aspirated and washed once with pre-cooled PBS. The PBS was discarded, 100 µL of lysis solution added and a pipette was used to agitate and thoroughly lyse cells. After centrifugation at 12,000 × *g* for 5 min at 4 °C, SOD activity in the supernatant was determined with a total superoxide dismutase assay kit (Beyotime, Shanghai, China).

To determine intracellular ROS concentrations, DCFH-DA (Beyotime, Shanghai, China) was diluted with serum-free culture medium at a ratio of 1:1,000. Cells were collected, suspended in diluted DCFH-DA, and incubated in a 37 °C cell culture incubator for 20 min, with inversion and mixing every 3 min to promote contact. Thereafter, cells were washed 3 times with serum-free cell culture medium to remove residual DCFH-DA, then evaluated using a flow cytometer.

### Western blot

Cells were treated as described above. Additionally, a potent ferroptosis inhibitor Liproxsrain-1 (25 nm, MCE, Shanghai, China) and ferroptosis inducer Erastin (10 μm, MCE, Shanghai, China) were used to treat bMECs for 6 or 9 h. Then, cells were collected and lysed for 5 min and the resulting suspensions were collected and centrifuged at 12,000 × *g* for 15 min at 4 °C. Total protein concentration was determined with a BCA protein assay kit (Beyotime, Shanghai, China), according to the manufacturer’s instructions. The sample was mixed with SDS-PAGE protein loading buffer and boiled for 10 min. After being blocked with 5% nonfat milk for 2 h, the membrane was incubated with primary antibodies for Nrf2, Keap1, HO-1, GPX4, ACSL4, S100A4, α-Tubulin, GAPDH, HSP70, TSG101, and CD63 overnight at 4 °C. Thereafter, it was incubated with secondary antibody HRP-conjugated goat anti-rabbit IgG (H + L) or HRP-conjugated goat anti-mouse IgG (H + L) for 1 h at room temperature. After washing with Tris-buffered saline, the membrane was developed using ECL reagents and visualized with a chemiluminescence system. Band density was assessed with Image J software (National Institutes of Health, Bethesda, MD, USA).

### Effects of EVs in murine mammary gland tissue

Twenty-four ICR mice (20 d of pregnancy) were housed in the laboratory animal room at the Experimental Animal Center of China Agricultural University. These mice were randomly allocated into 8 groups: Control; *K. pneumoniae* infection; *K. pneumoniae* + C-EVs; *K. pneumoniae* + M-EVs; *K. pneumoniae* + CM-EVs; C-EVs; M-EVs; and CM-EVs. Extracellular vesicles (including C-EVs, M-EVs and CM-EVs) and *K. pneumoniae* were injected into the mammary gland through the distal end of the nipple. The test was performed 5 d after parturition. Mouse pups were separated from female mice, and after 1 h, the experiment started. Female mice were anaesthetized with Zoletil 50, and the fourth pair of nipples was sterilized with 75% alcohol. With the aid of a stereomicroscope, the distal end of the nipple was excised with iris scissors. For the *K. pneumoniae* infection group, *K. pneumoniae* liquid (100 µL/papilla, 10^5^ CFU/mL) was slowly injected into the mammary gland with a micro syringe. For the EVs and *K. pneumoniae* group, injection of EVs was done first (100 µL/papilla, 2 mg/mL), followed 2 h later by infection with *K. pneumoniae* (100 µL/papilla, 10^5^ CFU/mL), respectively. Moreover, all groups of EVs were only injected with EVs (100 µL/papilla, 2 mg/mL).

### Immunofluorescence of murine mammary gland tissue

At 12 h post-infection or after the latest injection, all mice were anaesthetized, euthanized, and then the fourth pair of mammary gland tissues were excised, fixed in 4% paraformaldehyde for 48 h, then embedded in paraffin and sectioned (4 µm). After dewaxing and dehydrating paraffin sections, the primer antibody (ZO-1, Occludin, GPX4, and Nrf2) with a 1:500 dilution was added to the tissue section and then incubated at 4 °C overnight. The tissue section was washed with PBS 3 times, then a secondary antibody [CoraLite594-conjugated Goat Anti-Rabbit IgG(H + L)] with a 1:200 dilution was incubated at room temperature for 1 h, then washed with PBS 3 times. DAPI staining solution was used to stain the nucleus, sections were dehydrated with increasing concentrations of alcohol, and finally sealed with neutral gum. Protein expression of ZO-1, Occludin, GPX4, and Nrf2 was observed under a fluorescence microscope.

### Statistical analyses

All data were analyzed by Student’s *t*-test or one-way ANOVA using SPSS 22.0 software with Bonferroni correction for multiple comparisons. Data were reported as mean ± standard deviation (SD). For all analyses, *P* < 0.05 was considered significant.

## Results

### Morphology and characteristics of milk-derived EVs

**Fig. 1:**
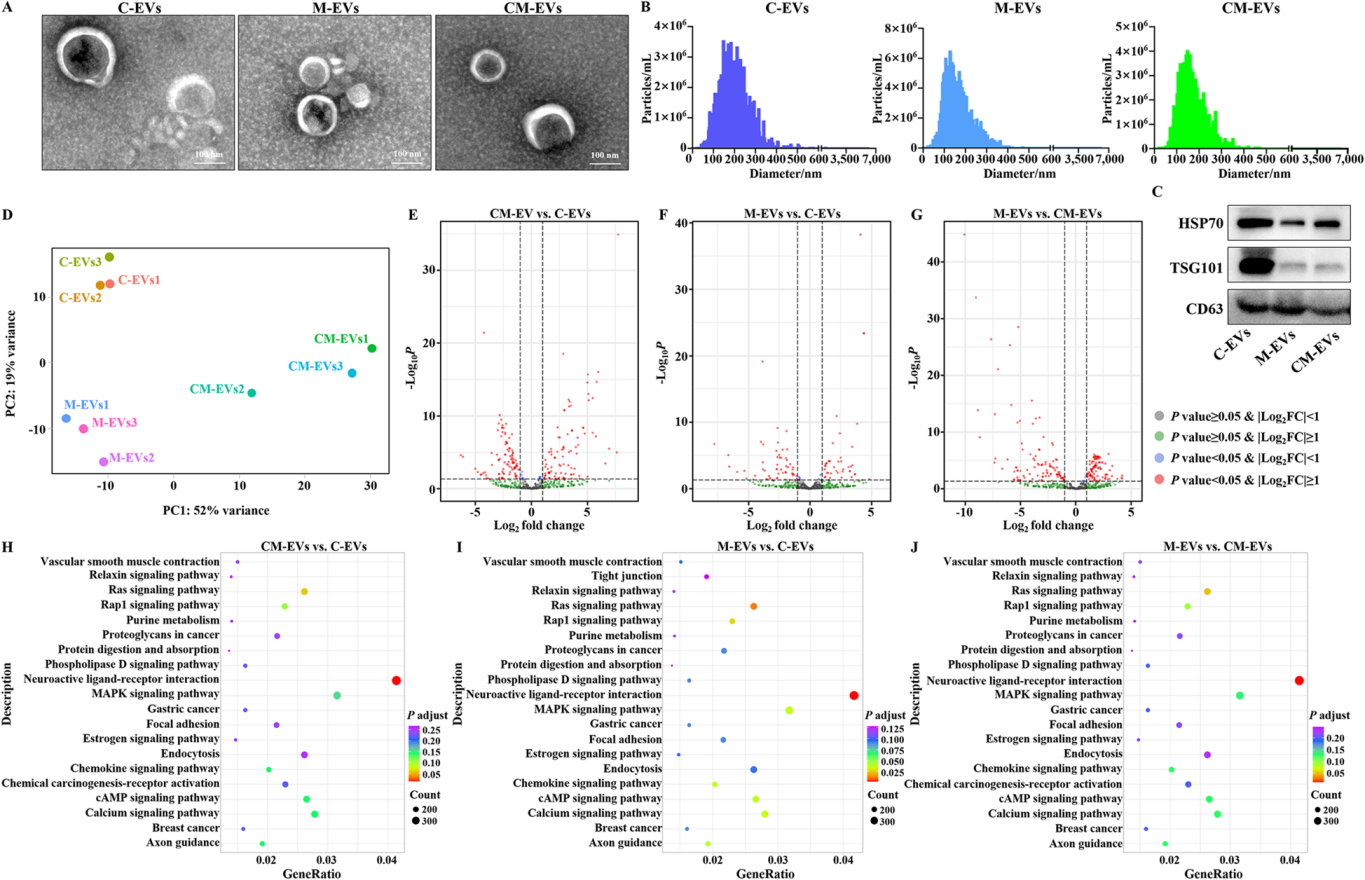
Identification, differential genes and sequencing analysis of extracellular vesicles (EVs) from 3 sources of bovine milk. **A** Transmission electron microscopy observation of EVs. **B** Measurement of EV particle diameter of bovine colostrum, mature milk and mastitis milk (defined as C-EVs, M-EVs and CM-EVs, respectively). **C** Detection of EV markers by Western blot. **D** PCA based on DESeq2. **E** Analysis of miRNAs in CM-EVs vs. C-EVs based on volcano maps. **F** Analysis of miRNAs in M-EVs vs. C-EVs based on volcano maps. **G** Analysis of miRNAs in M-EVs vs. CM-EVs based on volcano maps. **H** KEGG top20 enrichment pathway analysis of CM-EVs vs. C-EVs. **I** KEGG top20 enrichment pathway analysis of M-EVs vs. C-EVs. **J** KEGG top20 enrichment pathway analysis of M-EVs vs. CM-EVs

Three kinds of extracellular vesicles (C-EVs, M-EVs and CM-EVs) were obtained from colostrum, mature milk and clinical mastitis milk, respectively, by differential centrifugation. All 3 had a double-layer membrane (Fig. 1A). C-EVs, M-EVs and CM-EVs had particle diameters of 147.3, 174.6 and 139.7 nm, respectively (Fig. 1B). All EVs had expression of marker proteins HSP70, TSG101 and CD63 (Fig. 1C), confirming that they were EVs.

### MiRNA sequencing analysis of EVs

Principal Component Analysis (PCA) indicated that dispersion of EVs from the same source was low, whereas dispersion of EVs from disparate sources was high (Fig. 1D). Among 658 variables, there was a total of 102 upregulated miRNAs and 101 downregulated miRNAs in CM-EVs vs. C-EVs (Fig. 1E, Table S1); a total of 42 upregulated miRNAs and 57 downregulated miRNAs in M-EVs vs. C-EVs (Fig. 1F, Table S2); and finally, a total of 102 upregulated miRNAs and 122 downregulated miRNAs in M-EVs vs. CM-EVs (Fig. 1G, Table S3). Based on KEGG analysis, neuroactive ligand-receptor interaction was a significant difference in CM-EVs vs. C-EVs (Fig. 1H, Table S4). There was a significant difference for 8 pathways: neuroactive ligand-receptor interaction, Ras signaling pathway, Rap1 signaling pathway, MAPK signaling pathway, chemokine signaling pathway, cAMP signaling pathway, calcium signaling pathway, and axon guidance in M-EVs vs. C-EVs (Fig. 1I, Table S5). There were 2 pathways with significant differences between M-EVs and CM-EVs, including the Ras signaling pathway and neuroactive ligand-receptor interaction (Fig. 1J; Table S6). Therefore, differences between EVs within a milk source were very small, whereas EVs from disparate milk sources were distinctly different.

### *K. pneumoniae* induced oxidative stress and ferroptosis in bMECs

**Fig. 2:**
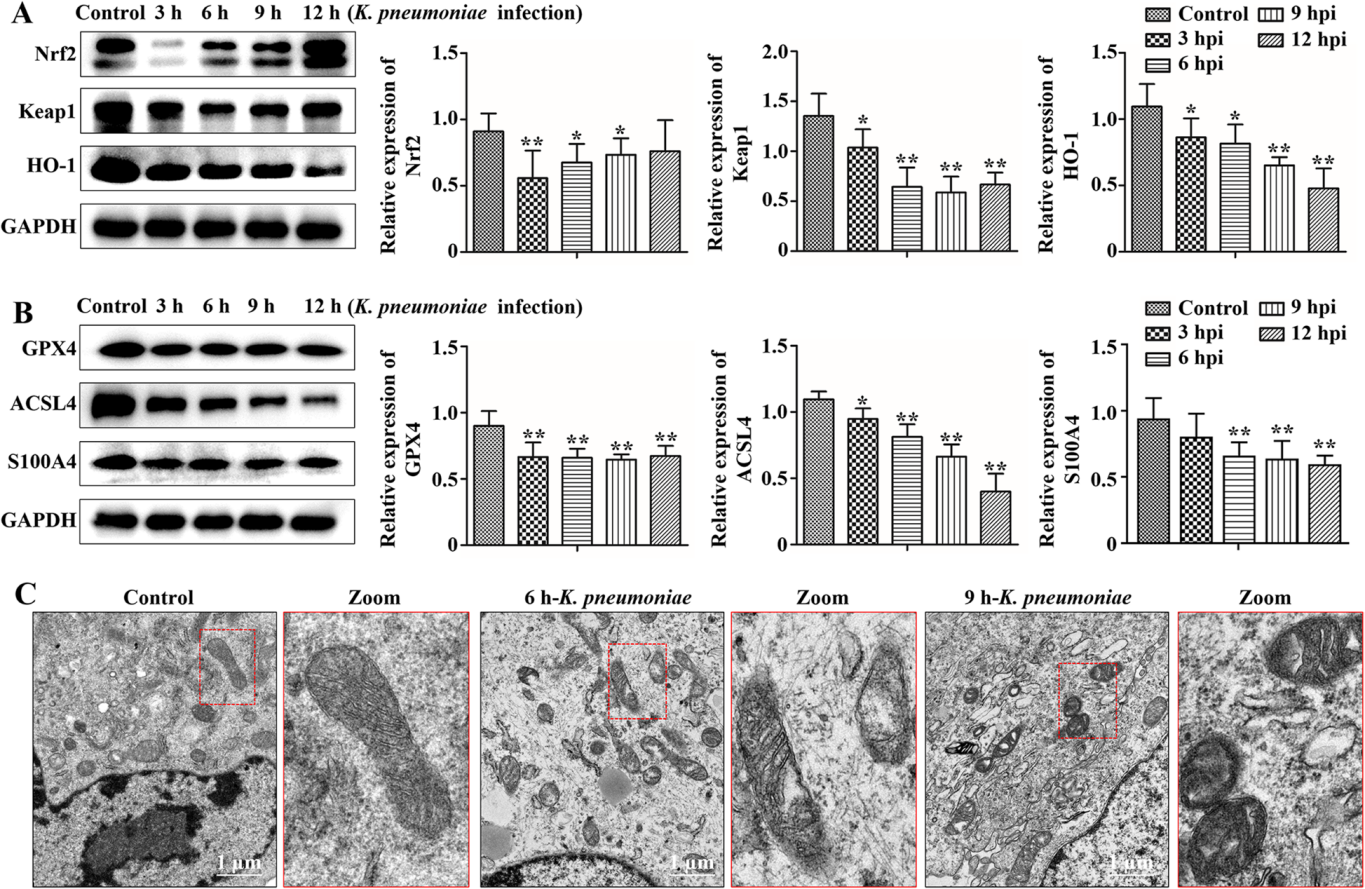
Protein expression in the Nrf2/HO-1 pathway and GPX4, ACSL4 and S100A4 in bMECs. **A** Changes of Nrf2, Keap1 and HO-1 in bMECs infected with *K. pneumoniae*. **B** Changes of GPX4, ACSL4 and S100A4 in bMECs infected with *K. pneumoniae*. **C** Ultrastructural observation of bMECs. Compared to the control group, ^*^P < 0.05 and ^**^P < 0.01

After 3, 6, 9, or 12 h of *K. pneumoniae* infection in bMECs, protein expression of Nrf2 had a trend of first decreasing and then increasing (*P* < 0.05, Fig. 2A), but it always remained lower than in the control group. However, protein expression of Keap1 and HO-1 decreased with increasing infection time and was significantly lower than the Control group (*P* < 0.05, Fig. 2A). Additionally, protein expression of GPX4, ACSL4 and S100A4 in bMECs, factors that regulate ferroptosis, was decreased in the *K. pneumoniae* infection group compared to the Control group (*P* < 0.05, Fig. 2B).

The ultrastructure of mitochondria bMECs was altered by *K. pneumoniae* infection. In the Control group, mitochondrial morphology was normal, and mitochondrial cristae and cristae were clearly visible (Fig. 2C). Conversely, after 6 or 9 h of *K. pneumoniae* infection, mitochondria appeared vacuolated, mitochondrial membrane density increased, and mitochondria became smaller (Fig. 2C), evidence that *K. pneumoniae*-induced ferroptosis in bMECs.

### EVs alleviated *K. pneumoniae*-induced oxidative stress in bMECs

**Fig. 3:**
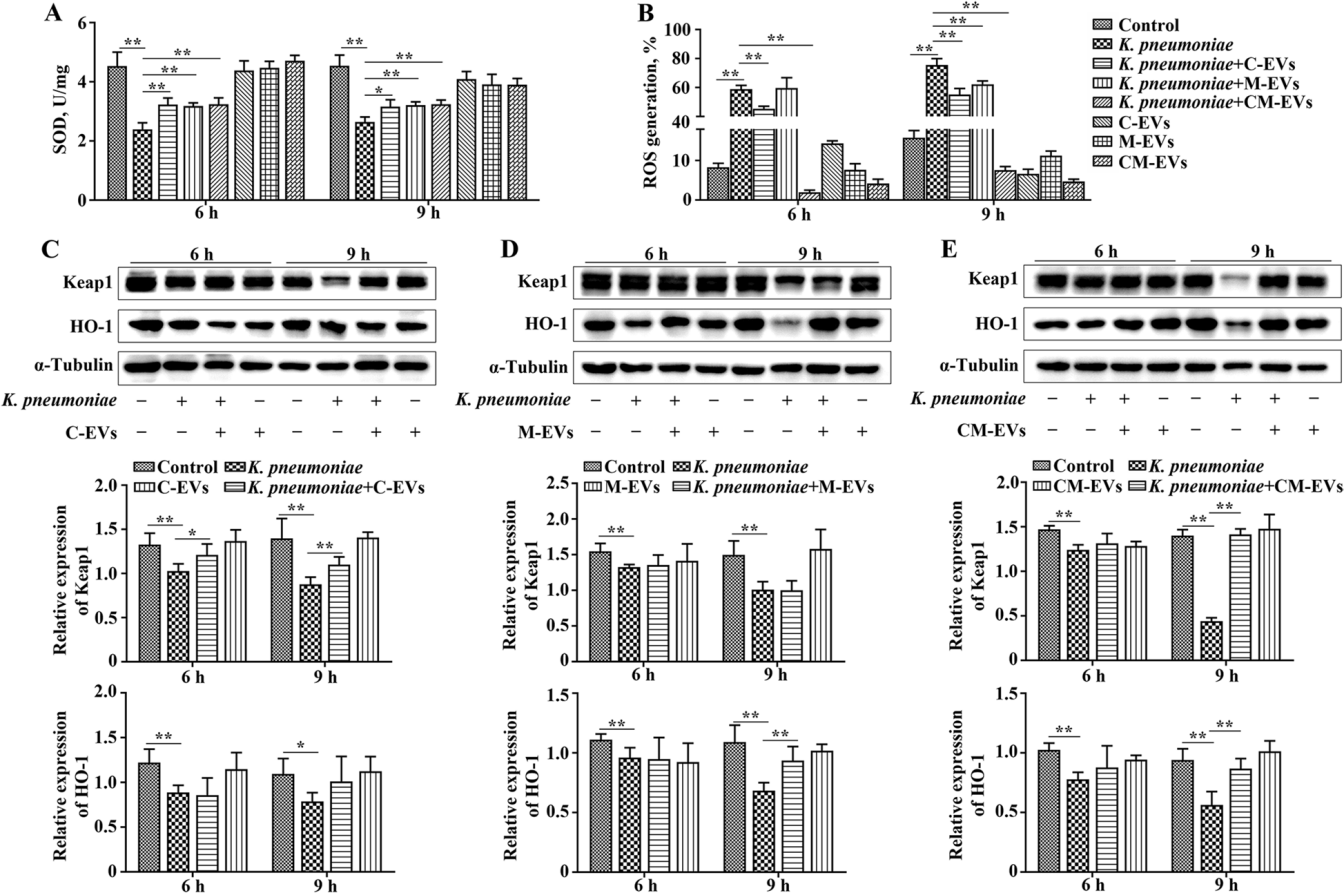
Extracellular vesicles (EVs) promote protein expression of Keap1 and HO-1 in bMECs infected with *K. pneumoniae.* **A** Changes of SOD activity in bMECs infected with *K. pneumoniae* or treatment with EVs. **B** Changes of ROS generation in bMECs infected with *K. pneumoniae* or treatment with EVs. **C**–**E** EVs from bovine colostrum, mature milk and mastitis milk (defined as C-EVs, M-EVs and CM-EVs, respectively) promoted protein expression of Keap1 and HO-1 in bMECs infected with *K. pneumoniae*. ^*^P < 0.05 or ^**^P < 0.01 is considered significant

After 6 or 9 h of *K. pneumoniae* infection, SOD activity in bMECs was decreased compared to the Control group (*P* < 0.01, Fig. 3A); however, addition of C-EVs, M-EVs, or CM-EVs to *K. pneumoniae*-infected bMECs increased SOD activity when compared to the *K. pneumoniae* infection group (*P* < 0.05, Fig. 3A). Addition of any of the 3 kinds of EVs in bMECs culture medium had no significant effect on SOD activity (Fig. 3A). After *K. pneumoniae* infected bMECs for 6 or 9 h, ROS generation in bMECs was increased compared to the Control group (*P* < 0.01, Fig. 3B). In contrast, adding C-EVs, M-EVs, or CM-EVs to *K. pneumoniae*-infected bMECs decreased ROS production compared to the *K. pneumoniae* infection group (*P* < 0.01, Fig. 3B). Furthermore, adding C-EVs, M-EVs, or CM-EVs to bMECs culture medium reduced ROS compared to the Control group at 9 h (Fig. 3B).

Infection with *K. pneumoniae* decreased protein expression of Keap1 and HO-1 in bMECs compared to the Control group at 6 or 9 hours post infection (hpi) (*P* < 0.05, Fig. 3C–E); however, pre-treatment with EVs prior to infection with *K. pneumoniae* increased Keap1 and HO-1 protein expression in bMECs as compared to the *K. pneumoniae* infection group at 6 or 9 hpi (*P* < 0.05, Fig. 3C–E). Additionally, adding EVs had no significant effect on protein expression of Keap1 or HO-1 at 9 h (Fig. 3C–E).

### EVs inhibited *K. pneumoniae*-induced ferroptosis in bMECs

**Fig. 4:**
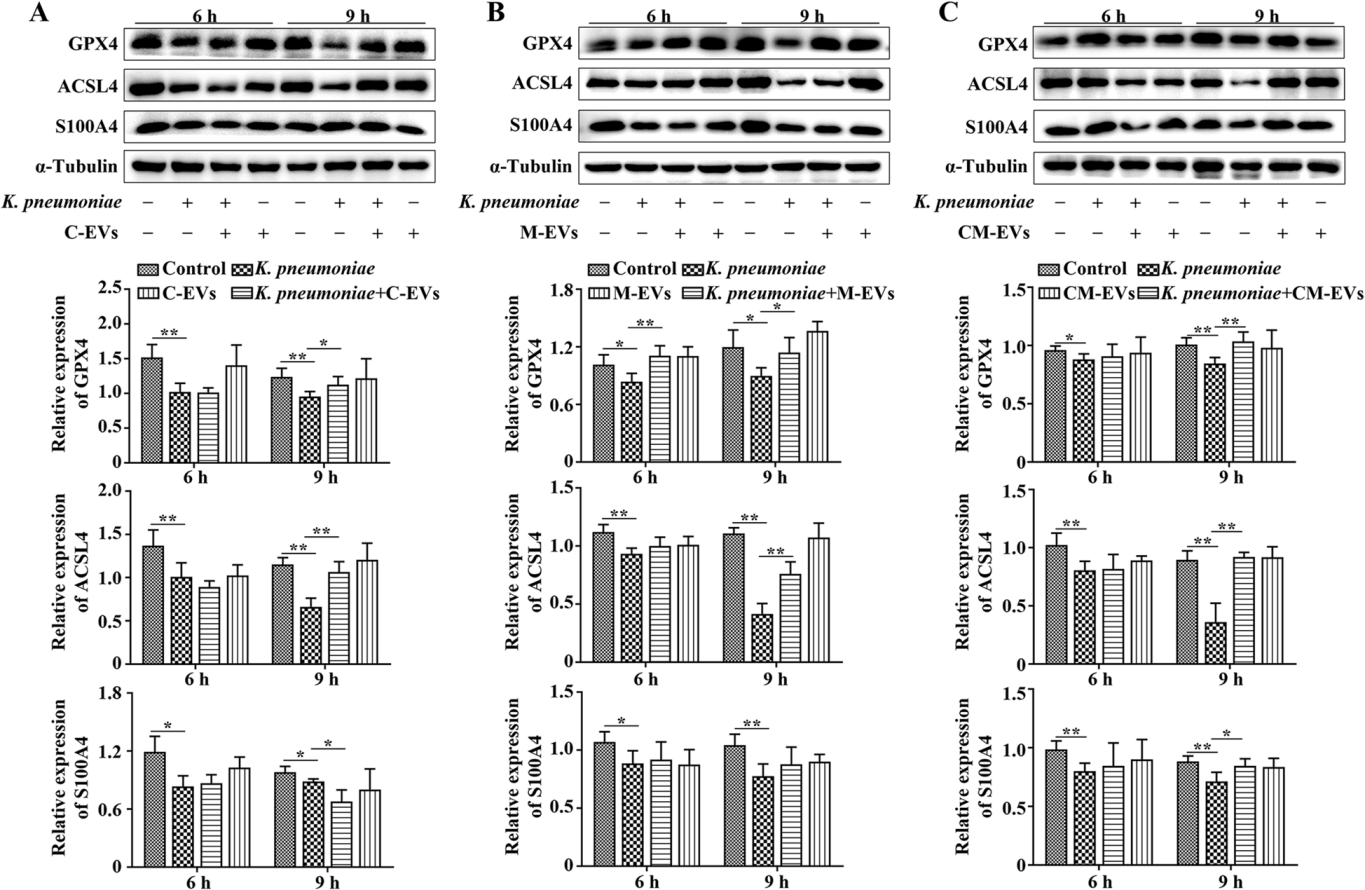
Extracellular vesicles (EVs) inhibit* K. pneumoniae* induced ferroptosis in bMECs. **A** C-EVs inhibits *K. pneumoniae* induced ferroptosis in bMECs. **B** M-EVs inhibits *K. pneumoniae* induced ferroptosis in bMECs. **C** CM-EVs inhibits *K. pneumoniae* induced ferroptosis in bMECs. ^*^P < 0.05 or ^**^P < 0.01 is considered significant

After 6 or 9 h of *K. pneumoniae* infection in bMECs, protein expression of GPX4, ACSL4 and S100A4 in *K. pneumoniae* infected bMECs was decreased compared to the Control group (*P* < 0.05, Fig. 4A–C). However, adding C-EVs, M-EVs, or CM-EVs to *K. pneumoniae*-infected bMECs increased protein expression of GPX4, ACSL4 and S100A4 when compared to the *K. pneumoniae* infection group (*P* < 0.05, Fig. 4A–C). Also, addition of C-EVs, M-EVs, or CM-EVs in bMECs culture medium had no significant effect on protein expression of GPX4, ACSL4 or S100A4 in bMECs compared to the Control group at 9 h (Fig. 4A–C).

### Inhibition of ferroptosis promoted Nrf2/Keap1 expression in *K. pneumoniae*-infected bMECs

**Fig. 5:**
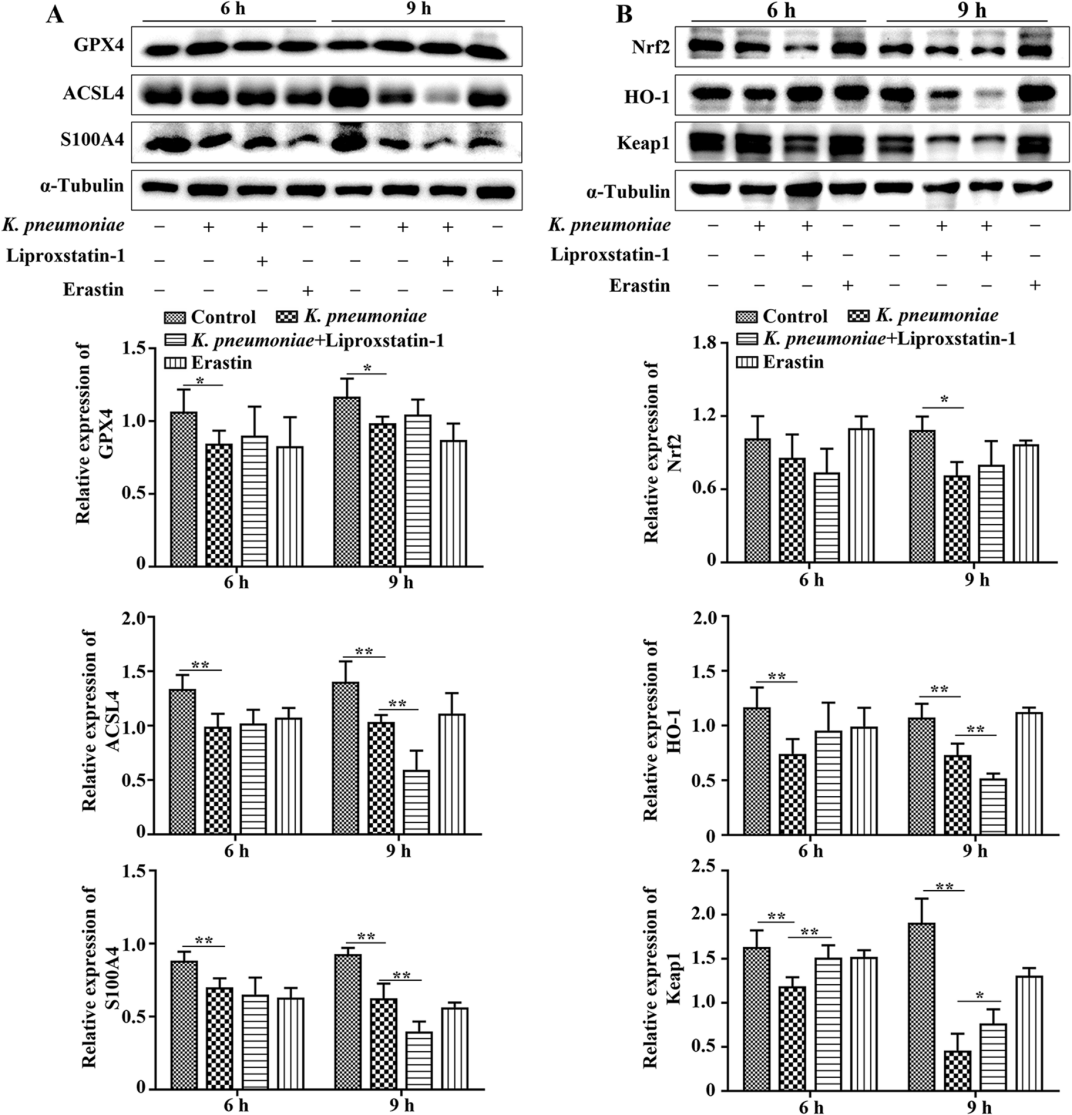
Inhibiting ferroptosis and promoting protein expression of Nrf2 and Keap1 in bMECs. **A** Liproxsrain-1 and Erastin regulated GPX4, ACSL4 and S100A4 expression in bMECs. **B** Liproxsrain-1 promoting protein expression of Nrf2 and Keap1 in bMECs. ^*^P < 0.05 or ^**^P < 0.01 are considered significant

After 6 or 9 h of *K. pneumoniae* infection in bMECs, protein expression of GPX4, ACSL4 and S100A4 was decreased compared to the Control group (*P* < 0.05, Fig. 5A). However, adding Liproxsrain-1 to *K. pneumoniae* infected bMECs increased protein expression of GPX4 and decreased ACSL4 and S100A4 protein expression in bMECs when compared to the *K. pneumoniae* infection group at 9 hpi (*P* < 0.05, Fig. 5A). In addition, after bMECs were treated with Erastin for 6 or 9 h, protein expression of GPX4, ACSL4 and S100A4 was decreased in bMECs compared to the Control group (Fig. 5A). Additionally, protein expression of Nrf2, Keap1 and HO-1 was decreased in *K. pneumonia-*infected bMECs after 6 or 9 h (*P* < 0.05, Fig. 5B). Adding Liproxsrain-1 (a potent ferroptosis inhibitor) to *K. pneumoniae*-infected bMECs increased protein expression of Nrf2 and Keap1 and decreased HO-1 protein expression in bMECs as compared to the *K. pneumoniae* infection group at 9 hpi (Fig. 5B, *P* < 0.05). After bMECs were treated with Erastin (ferroptosis inducer) for 6 or 9 h, protein expression of Nrf2 and HO-1 was increased in bMECs compared to the Control group (Fig. 5B).

### EVs alleviated *K. pneumoniae*-induced tight junction dysfunction in murine mammary tissue

**Fig. 6:**
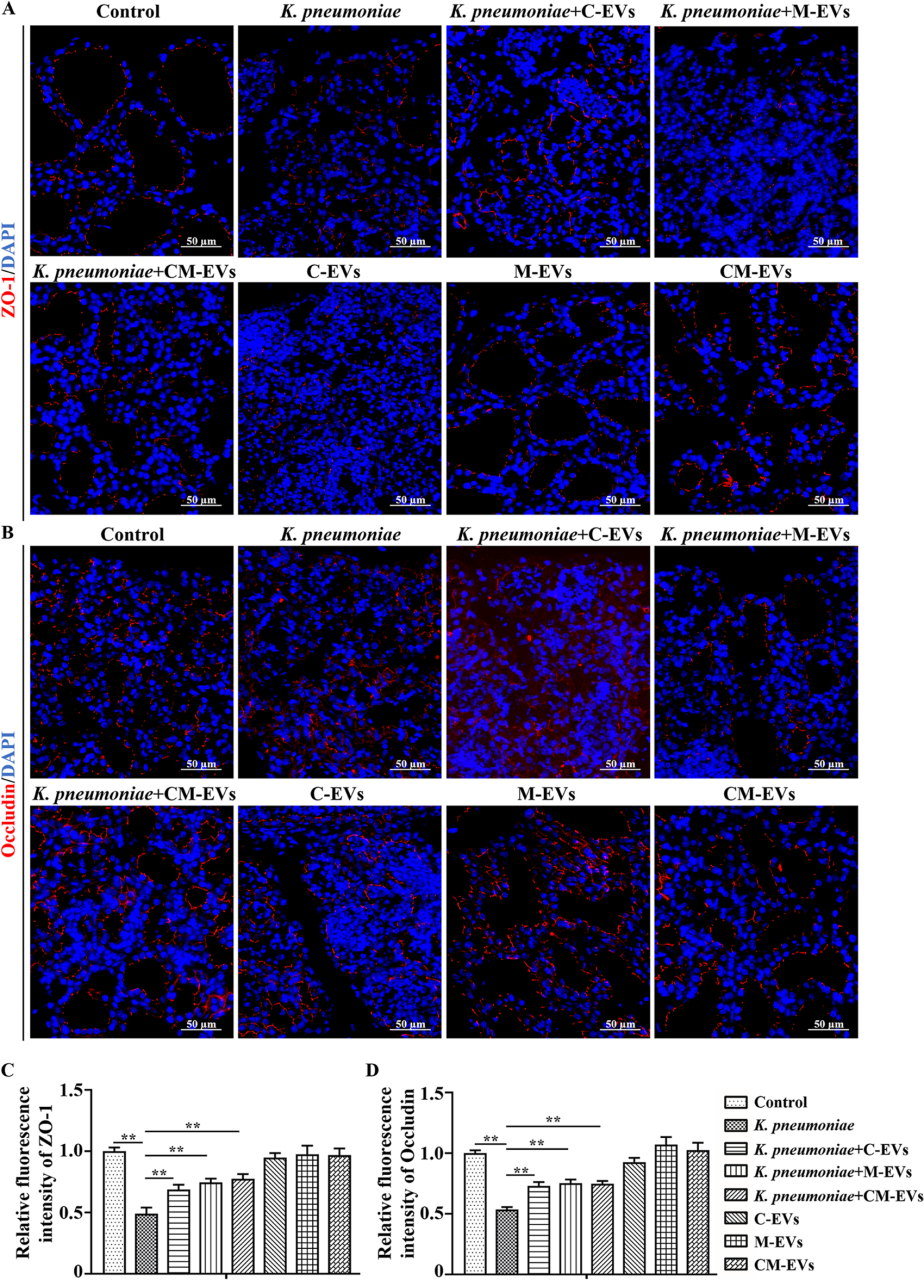
Immunofluorescence analysis of ZO-1 and Occludin expression in murine mammary gland. **A** and **B** ZO-1 and Occludin expression in murine mammary gland tissue. **C** and **D** Relative fluorescence intensity of ZO-1 and Occludin. ^*^P < 0.05 or ^**^P < 0.01 is considered significant

In murine mammary gland tissue of Control mice, ZO-1 and Occludin had strong red fluorescence, indicating that ZO-1 and Occludin proteins were both highly expressed (Fig. 6A–D). However, red fluorescence intensity of both ZO-1 and Occludin decreased in mammary gland tissue after *K. pneumoniae* infection for 12 h, indicating decreased expression of both proteins compared to Control group (*P* < 0.01, Fig. 6A–D). In contrast, pre-treatment with EVs before injection of *K. pneumoniae* increased red fluorescence intensity of ZO-1 and Occludin in mammary glands as compared to the *K. pneumoniae* infection group (*P* < 0.01, Fig. 6A–D). Furthermore, injecting only EVs had no significant effect on protein expression of ZO-1 or Occludin (Fig. 6A–D).

### EVs promoted expression of Nrf2 and GPX4 in *K. pneumoniae*-infected murine mammary tissue

**Fig. 7:**
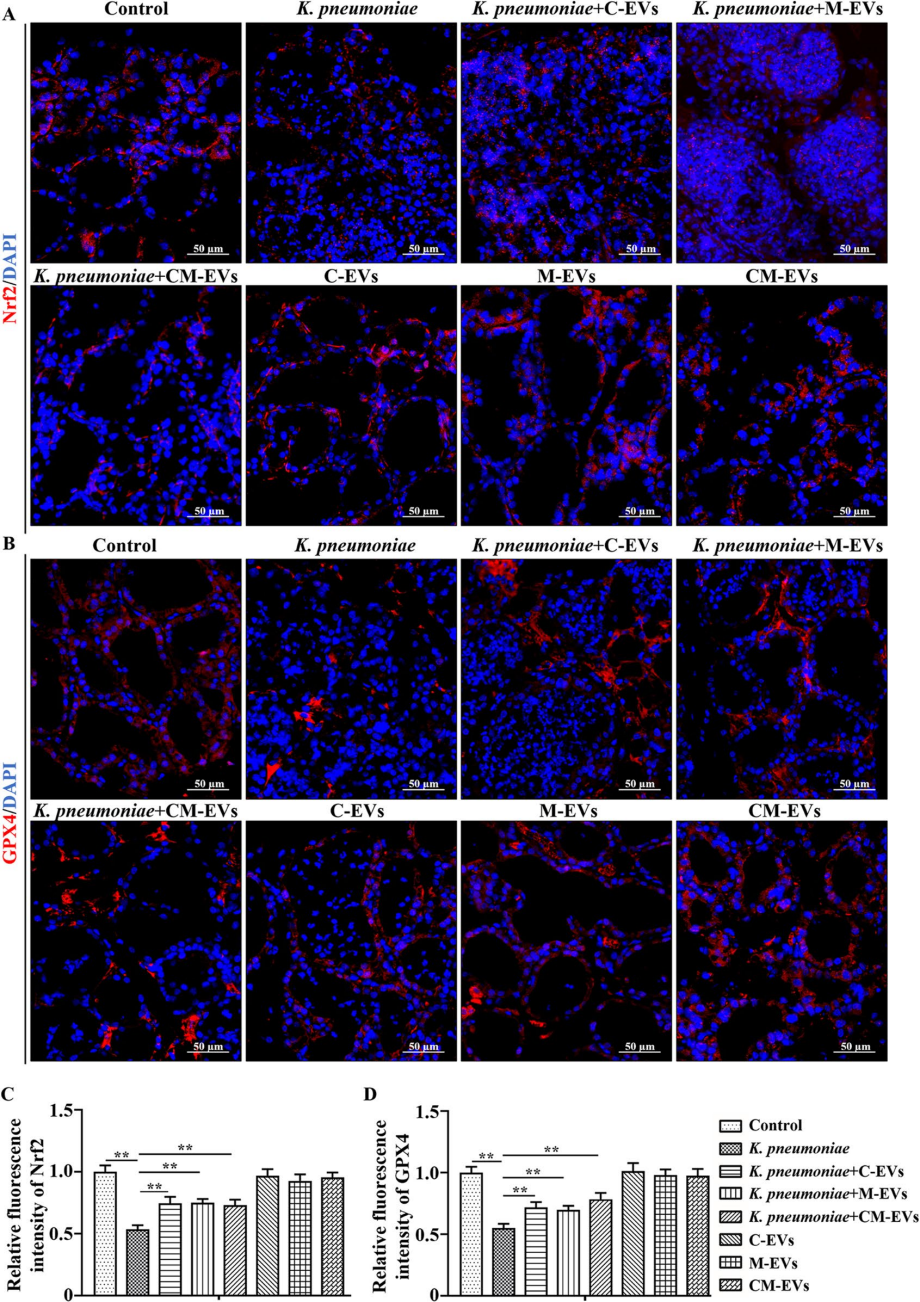
Immunofluorescence analysis of Nrf2 and GPX4 expression in murine mammary gland. **A** and **B** Nrf2 and GPX4 expression in murine mammary gland tissue. **C** and **D** Relative fluorescence intensity of Nrf2 and GPX4. ^*^P < 0.05 or ^**^P < 0.01 are considered significant

Both Nrf2 and GPX4 had a strong red fluorescence in the Control murine mammary gland tissue (Fig. 7A–D), indicating high protein expression. However, after *K. pneumoniae* infection for 12 h, red fluorescence intensity of both proteins had decreased, indicating that their protein expression was decreased compared to the Control group (*P* < 0.01, Fig. 7A–D). Injection of EVs into mammary glands before *K. pneumoniae* infection increased red fluorescence intensity of Nrf2 and GPX4 as compared to the *K. pneumoniae* infection group (*P* < 0.01, Fig. 7A–D). However, injecting EVs had no significant effect on expression of either protein (Fig. 7A–D).

## Discussion

In this study, 3 kinds of EVs, namely C-EVs, M-EVs, and CM-EVs were isolated from colostrum, mature milk and clinical mastitis milk, respectively. There were no significant differences among these 3 kinds of EVs regarding morphology, but particle diameter differed and miRNA sequencing confirmed genetic differences. Importantly, bovine milk-derived EVs alleviated the oxidative stress and ferroptosis induced by *K. pneumoniae* in bMECs and murine mammary tissues.

Milk-derived EVs, a kind of exosome, have characteristics in common with other exosomes, including bilayer membrane structure and particle diameters from 30 to 200 nm. Furthermore, they are generally similar in composition, as all contain CD81, CD63 and TSG101 [25, 26]. In the present study, C-EVs, M-EVs and CM-EVs all contained CD63, HSP70 and TSG101; however, expression of specific proteins in EVs from colostrum was higher than the other 2 sources. Additionally, based on a nanoparticle tracking assay, there were differences in particle diameters. Differences in the composition of the 3 kinds of milk-derived EVs were small. Milk contains mRNA, miRNA, ribosomal RNA, long non-coding RNA, etc., which are mainly distributed in EVs [25]. The miRNA in EVs has an important role. Tumor-associated macrophages-secreted taurine and tumor extracellular vesicle-delivered miR-181a-5p acted as a positive feedback loop to repress ferroptosis in prostate cancer [27]. Cardiac miRNAs enriched EVs from animals with chronic heart failure targeted Nrf2 downregulation and mediated crosstalk between the heart and the brain in regulation of sympathetic outflow [28]. The bta05208 pathway is a chemical carcinogenesis-ROS, whereas the bta04216 pathway is ferroptosis. There was no difference in these 2 pathways among all 3 kinds of EVs, implying that all 3 have potential to mediate oxidative stress and ferroptosis. Additionally, in terms of the enriched top20 pathways, the 3 kinds of EVs enriched almost identical pathways, implying similar biological functions.

Bovine milk-derived extracellular vesicles have important functions such as immune regulation, anti-bacterial infection and anti-oxidation. Iron-dependent programmed cell death, known as ferroptosis, is caused by unrestricted phospholipid peroxidation [29, 30]. Ferroptosis is primarily characterized by alterations in mitochondrial morphology, including condensation of the mitochondrial membrane accompanied by a decrease in size, rupture of the outer membrane and a reduction or disappearance of mitochondrial cristae. In the present study, mitochondrial shrinkage, increased membrane density, and mitochondrial crest lysis were observed in *K. pneumoniae* infected bMECs, suggesting *K. pneumoniae* may induce ferroptosis in bMECs.

GPX4 is a key antioxidant enzyme that eliminates hydroperoxides in the lipid bilayer and prevents accumulation of lethal lipid ROS, and also a key protein that inhibits ferroptosis [31, 32]. In this study, protein expression of GPX4 was decreased in *K. pneumoniae* infected bMECs; meanwhile, expression of S100A4, a ferroptosis-related protein that paralleled GPX4, and ACSL4 was decreased in *K. pneumoniae*-infected bMECs, suggesting that *K. pneumoniae* induced ferroptosis in bMECs. The ferroptosis induced by *K. pneumoniae* may aggravate bovine mastitis, as ferroptosis can regulate the inflammatory microenvironment through metabolic changes or secretion of related substances between microorganisms and host cells [33, 34]. Meanwhile, cells with ferroptosis can also recruit immune cells by releasing damage-related molecular patterns that induce generation of an inflammatory microenvironment [35, 36]. However, adding C-EVs, M-EVs, or CM-EVs to *K. pneumoniae* infected bMECs inhibited *K. pneumoniae*-induced ferroptosis. This may be associated with the antioxidant function of extracellular vesicles and the miRNA carried by extracellular vesicles; nonetheless, further investigation is required to elucidate the specific regulatory mechanism.

Ferroptosis occurs due to an accumulation of ROS, leading to lipid peroxidation, which exceeds the redox capacity of GSH and GPX4 [37, 38]. In this study, *K. pneumoniae* increased ROS and inhibited SOD activity in bMECs; this likely contributed to activating ferroptosis. Dysregulation of the Nrf2 axis, a key pathway regulating ROS generation, was observed in cells infected with *K. pneumoniae*, indicating that dysregulation of the Nrf2/Keap1 axis was also involved in *K. pneumoniae*-induced ferroptosis of bMECs. Under quiescent conditions, Keap1 restrains Nrf2 activity, whereas exposure to stress liberates Nrf2 from Keap1-mediated repression [39, 40]. HO-1 activity diminishes free heme concentrations and subsequently attenuates ROS production [41]. Similarly, SOD supplementation could reduce the risk of free-radical overproduction [42]. Ferroptosis involves lipid peroxidation and iron dependency under many types of regulated cell death processes in response to oxidative stress [43]. The response to oxidative stress is related to the interaction between GPX4 and autophagic degradation pathways, whereas activation of Nrf2 restrains ferroptosis [44, 45]. Infection of *K. pneumoniae* reduced Nrf2, Keap1 and HO-1 expression and enhanced ROS production in bMECs. However, pre-treatment with any of the 3 EVs significantly enhanced expression of Nrf2/Keap1 and attenuated ROS. Likewise, C-EVs and CM-EVs significantly augmented HO-1 expression in bMECs induced by *K. pneumoniae*. Moreover, EVs improved antioxidation anti-ferroptosis ability and restored cell barrier function in vivo. In mouse mammary gland tissue infection induced by *K. pneumoniae*, pre-treatment with C-EVs, M-EVs or CM-EVs augmented Nrf2/GPX4 and ZO-1/Occludin expression, diminished by *K. pneumoniae* infection.

## Conclusion

Three types of EVs (C-EVs, M-EVs, and CM-EVs) isolated from colostrum, mature milk, and clinical mastitis milk had similar double-layer membrane morphology consistent expression of marker proteins HSP70, TSG101, and CD63, but disparate particle diameters. Based on PCA, here was low dispersion of EVs from the same source but high dispersion from disparate sources, with significant differences in miRNA expression and KEGG pathways among EVs from colostrum, mature milk, and clinical mastitis milk, indicating distinct EV profiles among milk sources. *K. pneumoniae* induced oxidative stress and ferroptosis in bMECs, and 3 extracellular vesicles C-EVs, M-EVs, and CM-EVs were isolated from colostrum, mature milk and clinical mastitis milk alleviated the resulting oxidative stress and ferroptosis in bMECs and murine mammary tissues.

## Supplementary Information

Additional file 1: Table S1. Analysis of miRNAs in CM-EVs vs. C-EVs based on volcano maps.

Additional file 2: Table S2. Analysis of miRNAs in M-EVs vs. C-EVs based on volcano maps.

Additional file 3: Table S3. Analysis of miRNAs in M-EVs vs. CM-EVs based on volcano maps.

Additional file 4: Table S4. KEGG top20 enrichment pathway analysis of CM-EVs vs. C-EVs.

Additional file 5: Table S5. KEGG top20 enrichment pathway analysis of M-EVs vs. C-EVs.

Additional file 6: Table S6. KEGG top20 enrichment pathway analysis of M-EVs vs. CM-EVs.

## Data Availability

All data necessary to confirm the conclusions are included in this article. Datasets generated and/or analyzed during the current study are available from the author upon reasonable request.

## Abbreviations

ACSL4: Acyl-CoA synthetase long-chain family member 4
bMECs: Bovine mammary epithelial cells
C-EVs: Colostrum extracellular vesicles
CM-EVs: Clinical mastitis milk extracellular vesicles
EVs: Extracellular vesicles
GPX4: Glutathione peroxidase 4
HO-1: Heme oxygenase-1
Keap1: Kelch-like ECH-associated protein 1
*K. pneumoniae*: *Klebsiella pneumoniae*
M-EVs: Mature milk extracellular vesicles
miRNA: MicroRNA
Nrf2: Nuclear factor erythroid 2-related factor 2
PBS: Phosphate-buffered saline
ROS: Reactive oxygen species
S100A4: S100 calcium binding protein A4
TEM: Transmission electron microscope
